# University Day

**Published:** 1900-05

**Authors:** 


A Monthly Review of Medicine and Surgery.

EDITOR:

WILLIAM WARREN POTTER, M. D.

All communications, whether of a literary or business nature, books for review and exchanges should be addressed to the editor: 284 FRANKLIN STREET, BUFFALO, N. Y.

VOL. XXXIX.—LV. MAY, 1900. No. 10.

### UNIVERSITY DAY.

COMMENCEMENT OF MEDICAL AND PHARMACAL DEPARTMENTS.

THE fifty-fourth annual commencement of the Medical Department and the thirteenth of the Department of Pharmacy of the University of Buffalo, were held on Friday, April 27, 1900.

THE MORNING.

During the first part of the day, as usual, examinations were held by the curators. While this important office was being conducted in one part of the building, in another the arriving alumni were greeting and being greeted. Registration and payment of dues were next in order and, these being attended to, came reunions of classes, and informal visiting by the now rapidly arriving alumni. They were taken in charge by the reception committee, Dr. Nelson W. Wilson, chairman of the general committee, and Dr. Margaret S. Halleck, chairman of the women's committee, and escorted to the several rooms set apart for these meetings.

The classes of 1850, 1860, 1870, 1880 and 1890 got together, those of them that are left, and talked over old times and dug up out of the past reminiscences of the good old days when they were students and were passing through the vale of hopes and fears just as the fledgings in medicine were passing then through the same ordeal.

Officers of the class of 1870 were elected as follows: President, Dr. C. D. Kibler, Corry, Pa.; secretary and treasurer, Dr. L. W. Byam, Mumford, N.Y.

Officers of the class of 1880 were elected as follows: President. Dr. Ten Eyck O. Burleson, Bath, N.Y.; secretary, Dr. C. H. Woodard, Buffalo; treasurer, Dr. C. M. Daniels, Buffalo.

Officers of the class of 1890 were elected as follows: President, Dr. D. J. Tillotson, Rochester; vice-president, Dr. J. J. McAdam, Buffalo; secretary and treasurer, Dr. B. C. Johnson, Buffalo.

The classes of 1850 and 1860 elected no officers.

After the reunions luncheon was served at the Iroquois.

After the curators had completed the examinations of the class of 1900, they were entertained at luncheon by the faculty at the Buffalo Club.

THE AFTERNOON.

*Alumni Association Meeting.*

The twenty-fifth regular session of the Alumni Association was called to order by the President, Dr. William Warren Potter, in Alumni Hall, at 2.30 o'clock.

After the minutes of the previous session had been read and approved the treasurer, Dr. H. U. Williams, presented his annual report. It was in detail and showed :

| | |
|---|---|
| Receipts during the year from all sources . . . . . . . . | $546.52 |
| Expenditures . . . . . . . . . . . . . . . . . . . . . . | 544.25 |
| Balance on hand, April 27, 1900 . . . . . . . . . . . | $2.27 |

On motion the report was accepted and an auditing committee authorised. The president named Drs. Allan A. Jones and E. H. Ballou as such committee. The committee subsequently reported the treasurer's statement correct in every particular.

On motion the president was empowered to appoint a nominating committee. The chair named Drs. F. W. Barrows, Eli H. Long, and H. G. Matzinger as a nominating committee, who reported as follows :

President, Dr. Devillo W. Harrington, Buffalo, class of 1871

First vice-president, Dr. Frank H. Moyer, Moscow, class of 1872.

Second vice-president, Dr. A. W. Bayliss, Buffalo, class of 1889.

Third vice-president, Dr. C. S. Payne, Liberty, class of 1880.

Fourth vice-president, Dr. Mary J. Slaight, Rochester, class of 1880.

Fifth vice-president, Dr. Clarence King, Machias, class of 1885.

Secretary, Dr. T. H. McKee, Buffalo, class of 1898.

Treasurer, Dr. Herbert U. Williams, Buffalo, class of 1889.

Trustee for five years, Dr. William Warren Potter, Buffalo, class of 1859.

Executive committee, Dr. Albert T. Lytle, Buffalo, class of 1893, chairman; Dr. Grover W. Wende, Buffalo, class of 1889, and Dr. Abram T. Kerr, Buffalo, class of 1897.

On motion, the secretary was instructed to cast the ballot of the association for the group of officers nominated. The secretary complied and the foregoing were declared elected officers for the ensuing year.

The president then delivered his annual address, the subject being Metrorrhagia and the menopause.

It was discussed by Drs. Hayd, Mann and Frederick.

Dr. Maud J. Frye next read a paper on The symptomatology of early ectopic gestation. It was discussed by Drs. Frederick, Mann, Charles A. L. Reed, of Cincinnati, and Hayd.

Dr. Grover W. Wende followed with an interesting talk on certain skin diseases, illustrated by a large number of most excellent stereopticon views.

Dr. L. A. Weigel, of Rochester, closed the proceedings with an informal talk in the Diagnostic value of the *x*-ray, illustrated by the stereopticon.

The JOURNAL will publish these papers in a subsequent issue.

At 5.30 p.m. the meeting adjourned *sine die.*

### THE EVENING.

#### *Commencement Exercises.*

Owing to the alterations going on at Music Hall the commencement exercises were held at Central Presbyterian Church. In the absence of the Chancellor, Hon. James O. Putnam, the degrees were conferred by the Vice-Chancellor, George Gorham, Esq.

At precisely 8 o'clock, a large audience having already filled the spacious church, the procession headed by the vice-chancellor, Mr. Gorham, and the dean of the faculty, Dr. M. D. Mann, marched into the auditorium to inspiring music and took their places, the officials on the platform, and the graduates in front of the same.

The following order of exercises were then observed :

After music by the orchestra, Rev. George B. Richards, rector of the Church of the Ascension, offered prayer.

Dr. M. D. Mann, dean of the faculty of the medical department, presented to the vice-chancellor the candidates for the degree of Doctor of Medicine. The Hippocratic oath was administered and degrees were then conferred upon the following :

### CLASS IN MEDICINE.

John Howard Acheson,

James Philip Barr,

Theodore V. Bauer, Ph. G.,

Hugh Ria Brownlee,

Charles Milton Burdick, A.B.,

James H. Carr,

Charles Thomas Crance,

Wellington A. Crofoot,

Rollin Otis Crosier,

Edward D. Gibson,

George Wesley Gorrill,

Edwin R. Gould,

George W. Grabenstatter,

Frank L. Grosvenor,

Nettie C. Heintz,

Burt Hibbard,

Edward W. Heim,

Leon R. Iutzi,

William H. Jessup,

Edward W. Jones,

Bernard W. Junge,

David J. King,

Loretta L. Knappenberg,

Edgar R. McGuire,

Charles H. McVean,

Eva Viola Mead,

Raymond F. Metcalfe,

Mary E. Newman,

Luther Conklin Payne,

Minnette Pratt Petrie,

Frederick A. Pitkin,

Augustus G. Pohlman,

William Fred Powers,

David Hiram Ransom,

Frank Howard Ransom, A.B.,

Charles Lawrence Schang,

Eli Shriver, Jr.,

Alton L. Smiley,

Ellis W. Storms,

Ora Clay Swift,

Seth Newland Thomas, A.B.,

Beatrice A. Todd,

Alexander M. Troup,

Anna Warnecke,

George Erwin Welker,

Charles Sumner Wilson,

Ralph H. Willse,

Jacob B. Young.

Dr. John Parmenter, secretary of the faculty, announced the honors for the graduates in medicine. Augustus G. Pohlman, son of Dr. Julius Pohlman, of Buffalo, stood at the head of his class for the four years of the university course. His average standing for the entire four years was 91.6. Charles Sumner Wilson was second, with a standing of 91.1. Charles Milton Burdick was third, with a standing of 91, and the fourth was Mary F. Newman, whose average was 90.1.

Dr. John R. Gray, secretary of the department of Pharmacy, called the roll of candidates for the degree of graduate in pharmacy. The candidates were presented to the Chancellor by Dr. Willis G. Gregory, dean of the department, and degrees were conferred upon the following:

### CLASS IN PHARMACY.

Merritt L. Albright, John Ballagh, Roscoe Henry Bard, Louis A. Bradley, Francis Maxon Brimmer, Marion Frank Brzezicki, Rolland Andrew Chandler, Lee H. Cotton, Edward Marvin Cummings, Allen Corbin Day, Charles Nathan Dean, Harley Edwin Dowman, George Francis Feries, Erwin L. Fish, Fannie L. Fish, Willis Bryan Fitch,

William V. Gale, William J. Gram, Jr., Reynold A. Janke, George Burgess Jenkins, Charles Leo Keenan, Jasper Kobler, Howard E. Lane, Charles Forrest Larzelere, James Hugh McAdam, Charles Lawrence McLouth, Austin Charles Marble, Willis Lee Merkley, Lee William Miller, Jesse Monroe Parker, Clarence Newton Reese, James Clark Spaulding, Jr., Walter Erwin Strong, Arthur Gordon Sortore, Cyrus Eugene Sunderlin, Wells Damon Walrath, Jesse P. Wetmore, Paul Skinner Whedon, Lauren Pettebone Young.

The diplomas of Harley Edwin Dowman, James Hugh McAdam, and Willis Lee Merkley, were withheld until they attain their majority.

Honors and prizes were then announced by Dr. Gregory, as follows: Merritt L. Albright was first, with a standing of 93.9 for the entire course. He was also awarded the William H. Peabody prize of $50. Harley E. Dowman was second, with a standing of 93.3. Jesse Parker Wetmore was third, with an average of 90.2. Fannie L. Fish was fourth, with a standing of 86.6. Charles McLouth came fifth and last on the list, with a standing of 85.7.

In the junior class, the faculty prize of $25 was awarded to Ernest Chase Holt, who stands at the head of his class, with a percentage of 95.5. Second is Rudolf C. Miller, with a standing of 94.9. Third is William C. Achilles, whose average is 93.5. Fourth is Harry C. Rider, with an average of 92.72. Fifth and last is George Stoll, with an average of 92.68.

Dr. Charles A. L. Reed, of Cincinnati, O., was then introduced and delivered the address of the evening. Beginning with a graceful exordium, he asked sententiously and pertinently, what would be the condition of the world if there were no medical profession? He then gave a sketch of folk-medicine, putting himself in the imaginary attitude of a professor addressing his class, all being believers in this strange admixture of superstition, ignorance and witchery. The treatment of the sick by the folk-doctor was about as it is now by the charlatan, Eddyist, Indian medicine man and trance medium—all of which the learned speaker applied with force and clearness.

Dr. Reed then gave an interesting chronological résumé of the epochs of medicine and sketches of the masters, beginning with Hippocrates, and including the time of Theophrastus, Galen, Avicenna, Harvey, Jenner, and McDowell, showing the origin, descent, and succession of legitimate medicine which alone has any claim upon an intelligent people.

In an eloquent peroration he addressed a few words to the graduates, which brought to a close one of the most entertaining, finished and instructive addresses we have listened to in many a day. It abounded in learning, wit, sarcasm, pathos and rhetoric; in metaphor and period; and has rarely been equalled for originality of thought or wholesomeness in its lessons.

THE BANQUET.

At the conclusion of the graduating exercises the faculty, alumni, and invited guests repaired to the Iroquois where the 25th annual dinner was served. Seats were taken at 10.25 o'clock in the spacious dining room at tables tastefully decorated and where covers were laid for 150 persons, every chair being occupied. The menu was well prepared and the service was of that sort which makes the Iroquois famous. Good music helped to enliven the occasion and a goodly number of women lent attractiveness to the scene. After an hour and a half spent in eating the dinner, the intellectual feast began.

Dr. William Warren Potter, president of the association, acted as toastmaster and inaugurated the speech-making with the following remarks:

He said the executive committee had asked him simply to preside, but he found himself loaded with the responsibility of conducting the ceremonies. Taking for his text the figures on the menu, 1875-1900, he continued:

TWENTY-FIVE YEARS!

What memories such a period of time enkindles! What trials it suggests! What triumphs it records! What disaster it engulphs! What achievements it makes possible !!

And yet 'tis but a breath. With a touch of the INFINITE it vanishes into eternity, fading "like a wreath of mist at eve" as millions of similar periods have done, "to darkle in the trackless void" made and ever making by remorseless time.

Twenty-five years !!

Within that period a child is born, passes through infancy, childhood and youth, goes through college, takes his place in community, and begins his life work. This, in brief, is the history of young individual life. In national life it is much the same. Territories are peopled, states are formed and before we realise it, senators and representatives in congress are debating over systems of education, methods of internal improvement, and other great problems of statehood for the new commonwealth.

Twenty-five years ago, when this association was organised, the University, whose 54th annual commencement we have witnessed tonight, was located well up-town at Main and Virginia Streets, almost on the border of a city of 100,000 inhabitants. Now on the summit of yonder hill at High Street, stands a magnificent building, located near the center of a prosperous city, the second in importance within the state, with 400,000 people who are busy with manufactures, arts, commerce, science and all the varied and various interests that concern this progressive period; and, are, withal at this very moment preparing to entertain within the next year all the nations of the western hemisphere, who will assemble here at a great exposition.

A quarter of a century ago there were seven professorial chairs embracing the principle departments of medicine, with, perhaps, a few clinical assistants; now, behold a great college, with its 70 professors, teachers and instructors. Then there were no laboratories of biology, bacteriology, pathology, physiology or chemistry, or perhaps only the faintest semblance of the two latter. Now all these are in full swing under the best of teachers.

In those days clinical teaching had not reached its present state of perfection; indeed, the didactic method was paramount, and the dictum of the professor was the pupil's authority. Now, bedside instruction constitutes the larger and better portion of the curriculum. The course then was of two years' duration, with no uniform requirements of preliminaries, and no license by the state was demanded. The course now is of four years and graded, the preliminaries must equal the acquirements of a high school graduate, and there is separate state examinations for license to practise.

Finally, in those days there were no women pupils and no alumnæ, while now there are more than 100 of the latter, who are well represented here this evening, every one a credit to Alma Mater.

It must not be inferred from what I have said that Buffalo University Medical College was not in those days a strong, well-equipped school, for, in reality, it was one of the best and fully abreast of the period in its methods of instruction. It was founded by some of the ablest men in the country, and the founders have been succeeded from time to time as they disappeared from the scene, by men of great learning, skill and profundity. If they did not ask as much of candidates for medical degrees as your present teachers, it was because nowhere was as much required as in this busy bustling age of iconoclasm—this iron age of positivism.

For obvious reasons, I may not speak of the individual members of the present teaching body. Many of them are grouped around these tables and within the sound of my voice. They are well known to you all; they have your confidence and esteem, as they have that of the citizens of this community. They are distinguished among the profession of this country as teachers and practisers of the various sciences and arts pertaining to medicine and surgery; indeed, their fame is not limited by the borders of American shores, but has gone abroad wherever legitimate medicine is recognised throughout the world.

The class of 1900 is fortunate, most fortunate, in the choice of its environment for entrance into the most humane and ennobling of the learned professions.

If, then, I may not speak individually of your faculty, I trust I may be pardoned for making personal reference to one of the number, one who is loved and respected by you all, as he is also by this entire community, one who has been greviously afflicted during the past few weeks with direful sickness, but who, happily, is now to all appearances on the high road to speedy recovery. I ask you, one and all, to rise in your places and drink his health, sending him words of greeting and best wishes for his early recovery, and for a long and prosperous life. Ladies and gentlemen, I pledge you the health of Professor Roswell Park.

The response was prompt, the audience rose en masse and amidst rousing cheers, flying napkins, and rattling glasses, the health of Dr. Park was eagerly drunk.

The toastmaster, paying little heed to the printed rotation, then announced the sentiments and called upon the speakers in the following order of sequence :

Alma Mater, . . . . . . . . . . . . . . . . . . . . . . . Dr. Charles G. Stockton.

" He thought as a sage, though he felt as a man."

The Class of '00, . . . . . . . . . . . . . . . . . . . Dr. William Fred. Powers.

" Think naught a trifle, though it small appear."

The Physician Idealized, . . . . . . . . . . . . . . . Dr. Cora Billings Lattin.

" It were a journey like the path of heaven,
To help you find them."

Preaching and Practising, . . . . . . . . . . . . . . Rev. George B. Richards.

The Pan-American, . . . . . . . . Hon. William I. Buchanan, Director-General.

" Lest men suspect your tale untrue,
Keep probability in view."

The Ohio Idea, . . . . . . . . . . . Dr. Charles A. L. Reed, Cincinnati, Ohio.

" There is occasions and causes why and wherefore
in all things."

Prof. Stockton, in response to the sentiment, "Alma Mater," pointed out that while the average resources of the medical schools of the country are not great, the University of Buffalo is exceptionally well equipped in many respects. In spite of that fact, however, the University needs help, and he appealed to the new graduates to help it all they could and urged them all to unite with the Alumni Association and to take an active part in its proceedings. It was a bright speech, eloquent, witty and serious by turns.

Dr. William Fred Powers, responding to the sentiment "The Class of '00" delivered a neat appreciation of the efforts of the faculty which, he said, had had the result that the members of the class just graduated knew more about the practice of medicine than anyone else in the world.

Extremely bright and clever was the response of Dr. Cora Billings Lattin to the toast "The Physician Idealised." She appealed for wider recognition, by the male members of the profession, of the value and need of the woman physician and pointed out that besides filling a real need in the life of suffering womankind, the woman doctor could do much towards teaching medical ethics of many characteristics which are undesirable.

Rev. George B. Richards was asked about ten minutes before he stood up to speak, to respond to the toast "Preaching and Practising." He readily assented and in a clever and witty impromptu speech made an appeal for the admission of the clergy into sick rooms. "If the preacher is a fool," he said, "keep him out, just as you would keep out a fool doctor, but recognise the fact that a wise clergyman can often be of real benefit to your patient."

Director-General Buchanan spoke on "Reciprocity in Medicine." He declared that the South American republics today offer the best field in the world for the American physician who can talk Spanish, and he told of efforts that are being made to effect an interchange of courtesies that will secure the recognition of American diplomas in those countries.

Dr. Charles A. L. Reed, of Cincinnati, chairman of the executive committee of the Pan-American Medical Congress, who followed Mr. Buchanan, confirmed his statement regarding the value of South America as a field for practice, and offered to give all those present such credentials as would insure them a foothold in any of the Latin-American republics. Then, in a witty reply to Mr. Richards, he pointed out that the danger of allowing ministers in the sick room was that they might induce the patient to try some of the patent medicines which the clergy are so fond of endorsing for advertising purposes.

At 2.10 a.m. the toastmaster thanked the speakers and the audience who had so ably responded to the sentiments of the evening and so patiently listened thereto, thus materially aiding the executive committee in its efforts to make the occasion interesting. He then declared the meeting adjourned.

PEN PICTURES OF THE MEETING.

One of the gayest of the gay throng was Dr. G. R. Hinman who is a sort of a double header. He got out in '70 and he came back and belonged to the class of '98, which has been variously termed "wild Indians" and "holy terrors." They were an irreverant gang and to them Dr. Hinman was "Pop" from the day he joined them. And he was one of the boys in spite of the luxuriant growth of dignity which flourished on his chin. The boys loved him because he was always willing to help them, and told them stories and possessed a never failing wit and humor. It was Dr. Hinman's famous malarial mot which loosened the screws in Dr. Stockton's dignity and started him laughing. Dr. Hinman had been practising in lumber camps and much of his work had been of a luetic character; when Dr. Stockton asked him one day: "How do you treat malaria in Michigan, doctor?" the dear old "Pop" answered mildly, but with conviction: "Iodide of potassium and mercury."

Dr. Hazen of '98, who was president of the Rotten Row Club in the senior year has grown a mustache since he has been at the General Hospital, and he is going to practise in the Adirondacks, too. Dr. Hazen was on the reception committee. He and Dr. J. S. Otto took trolley rides in the afternoon.

Dr. Conrad Diehl took his weather eye off of Fisher long enough to run in and register. Then he flurried out in pursuit of a boom, which looked like the one he has in the incubator.

The register showed up some mighty poor specimens of handwriting. The race for the prize was a tie between three signers. "Henry Dow, Buffalo," looked like it might have been written with a club-foot. Somebody "Higgins, Sanborn, N.Y.," and a careful examination of the signature has failed to solve the mystery of the initials, wrote like a man with his hands tied behind his back. "Wilson, Buffalo," looked as if it had been written with a cross-cut saw. What his first name is could only be guessed at. There were ink marks on the book, but they had no human semblance. Each of these gentlemen should carry a rubber stamp, or an identification card.

Dr. Henry Lapp, of Clarence, and Dr S. W. Wetmore got together during the day. That means a great deal, too, for each is a story-teller and full of reminiscences.

Dr. D. W. Harrington who was elected persident was nervous about it, it seemed. Anyway Dr. Diehl cast longing eyes at the tall man from out Main Street way, and mentally wished things might come to him as easily.

Dr. D. A. Currie, of Englewood, N. J., was there too. But the only thing which will make Dr. Currie miss a meeting will be his inability to be present. That's a sort of foolish remark but its true.

Dr. Frederick and Dr. Reed of Cincinnati, looked one another over like a couple of big chiefs about to engage in a tribal warfare. Instead, they got together in a tubal argument and cited so many cases of ectopic pregnancy, that a less prosaic outfit than a lot of doctors might have very naturally wondered if the majority of babies were born the old-fashioned way or through a tube.

Dr. Hayd when he discussed Dr. Maud J. Frye's paper on ectopic pregnancy made a few remarks which were eagerly listened to by the students present. There is much fascination about the subject which is so closely crowding cocaine for first place in popular favor.

Some day some one is going to print a story about Dr. C. A. L. Reed, of Cincinnati, and he will have no one to blame but himself, for he told it to a handful of doctors. Far be it from me to violate confidence, but if you happen to run across Dr. Reed, ask him to tell you about the time he went up in the mountains of Kentucky, to help out a poor and struggling country practitioner who had written him for operative help. He will tell you that he went up there and did half a dozen operations, was gone a week, got held up by a flood, and all for $300. Then ask him how he found out that that poor, innocent country doctor had collected about a $1,000 for the work and kept the balance. But ask him. He might not like to have the story made public. This can be told, however, for it only shows Dr. Reed's natural interest in the profession. A year later he met a native of the region on whose abdomen he had made his mark, and asked how the doctor was getting along: "Fine, fine," was the answer, "he's built him a new house."

The surviving members of the D. E. Society held a session in the college building to make preliminary arrangements for the semi-centennial meeting next April and to choose a member from the class of '00 for election next year. Only one member is admitted each year.

Dr. Cora Billings Lattin's speech at the dinner was one of the most graceful, most self-contained speeches ever delivered in Buffalo by a woman. And what is more she said something of importance and she said it all neatly.

The man who is running the Pan-American prelude and who will direct the whole affair, Hon. W. I. Buchanan, sat beside the toastmaster at the banquet. The other side of him was snuggled up to Dr. Reed. Mr. Buchanan told some Spanish and South American stories between courses which were mighty interesting. He has traveled all over South America and knows the native like a book. Some day a wise and discriminating administration will be looking for the proper man to represent the United States at an important European court and it need not surprise anyone to see that administration strike Buchanan's trail and follow it up. He's a big man, mentally, physically and diplomatically. *En passant*, he is going to announce the appointment of his medical director for the Exposition pretty soon, so you want to keep your weather eye on the lightning rods which have been hoisted by the profession.

Dr. Stockton made a mighty bright speech at the dinner and why should he not? Did he not sit between Dr. Mann and Dr. Harvey R. Gaylord?

When you want spiritual help hoist your danger signal and steer for Rev. G. B. Richards, rector of Ascension Church. There's a minister who is a man with a heart, and not a square-cut coat full of prayers. Mr. Richards spoke at the dinner. He did not make a speech at all; he just spoke and he told the doctors some truths about preaching which they will remember. He also remarked a few things about medicine which were correct. The nice thing about this minister is that he has none of that offensively protuberant religion against which one so often bumps, at dinners which are unfortunate enough to have secured the services of a fool minister as a speechist. He strikes one as a man whose religion is of the broad-gauge character; a man who would go into a sick room and carrying with him cheer, comfort, rest and smiles, rather than a hell-and-damnation face and a God-have-mercy-on-your-poor-wicked-soul prayer. Can you imagine a man who publicly says that there are "fool ministers as well as fool doctors," being anything but a help and a comfort in a sick room. Mr. Richards tells stories, too, and he tells them well. He is a publicist and a preacher; that much he admits. He is likewise a peach.

The most eloquent event at the dinner was the standing toast drunk to the speedy restoration to perfect health of Dr. Roswell Park. And at the conclusion of the toast a dozen glasses were shattered. Count up on the fingers of one hand the men in this city who command such affectionate respect.

The newspaper men gave thanks to the executive committee with good cause. They had a table to themselves where they could do their writing, where they could see and hear. And this was their first experience of the kind at a medical dinner—and for that matter at most of the other public dinners in this city. The space which the newspapers gave the event showed that they appreciated the little attention which did not put any one out and made the newspaper men feel as if they were welcome as they surely were.

